# Engagement and Retention in Precision Public Health: A Cascade Analysis of Two Cluster Randomized Trials

**DOI:** 10.1159/phg/aenag004

**Published:** 2026-09-09

**Authors:** Jushawn Macon, Ali Lateef, Audrey Aton, Nina Smock, Patricia Salyer, Thuelfaqar Rammaha, Mary C. Politi, Laura J. Bierut, Li-Shiun Chen, Alex T. Ramsey

**Affiliations:** a Division of Biology and Biomedical Sciences, Washington University School of Medicine, St. Louis, MO, USA; b Department of Psychiatry, Washington University School of Medicine, St. Louis, MO, USA; c Alvin J. Siteman Cancer Center at Barnes-Jewish Hospital, Washington University School of Medicine, St. Louis, MO, USA; d Bursky School of Public Health, Washington University, St. Louis, MO, USA

**Keywords:** Participant engagement, Behavior change interventions, Genomic research, Clinical trials, Tobacco treatment, Lung cancer screening

## Abstract

**Introduction:**

When research fails to reach and engage all populations who might benefit from study findings, it can compromise scientific validity and ultimately health equity. Few studies have systematically examined factors driving study engagement through longitudinal intervention research. This project examined sociodemographic, geographic, and structural influences on engagement and attrition across the participation “cascade” (outreach, enrollment, and retention) for two large-scale multilevel precision medicine and precision prevention trials for smoking and lung cancer screening.

**Methods:**

Modified Poisson regression models were used to determine the factors associated with study engagement based on sociodemographic and geographical factors at each step in the cascade of participation, from initial outreach through retention at 12 months post-enrollment. Secondary analyses examined the cascade among the subset of patients who had active electronic patient portals and were approached via the portal.

**Results:**

A total of 24,366 patients were approached for participation. Race, social vulnerability index, insurance status, and distance from the study site were significantly associated with engagement at various points in the cascade. Black patients were more likely than white patients to be reached (48.9% vs. 47.1%; *p* = 0.022) and to complete eligibility screening (52.8% vs. 38.4%; *p* < 0.001), but less likely to consent to participate (56.7% vs. 69.8%; *p* < 0.001) and complete genetic testing (58.5% vs. 69.8%; *p* = 0.003). Patterns of engagement through electronic patient portal versus nonelectronic recruitment channels also differed by race- and place-based factors, with black patients being less likely than white patients to respond in the portal (8.8% vs. 15.5%; *p* < 0.001), and patients who reside farther from the study site being more likely to respond in the portal compared to those who live closer (14.9% vs. 12.5%; *p* < 0.001).

**Conclusions:**

These findings highlight the need for tailored, stage-specific engagement strategies to ensure representative participation in genomic and behavioral intervention research to advance the integration of genomics into public health practice.

## Introduction

Behavior change interventions are essential tools for public health, driving reductions in chronic disease risk and promoting sustained improvements in public health. These interventions seek to modify patterns across a range of behaviors, including tobacco use and lung cancer screening, and many have demonstrated efficacy in controlled research settings [1]. However, understanding their effectiveness and scalability in real-world settings with patients, community members, and other potential intervention recipients is often limited by participant disengagement and attrition in clinical trials.

High participant attrition poses significant methodological challenges and equity-related concerns. When disengagement is driven by nonrandom factors, it can introduce systematic bias by altering the study sample, reducing statistical power, and limiting the generalizability of findings [2]. In the context of interventions targeting lung cancer screening and tobacco treatment, systematic disengagement risks magnifying existing inequities by disproportionately excluding populations most affected by disease burden. Despite this threat, much of the existing literature remains largely descriptive, offering limited insight into the multilevel and dynamic factors that shape engagement and disengagement over time. Understanding participant disengagement over time is key to designing pragmatic clinical trials [3], developing participant-centered retention strategies [4], and ultimately enabling the equitable dissemination and implementation of evidence-based interventions [3, 5].

Participant disengagement is particularly pronounced in behavioral intervention research, with retention, defined from enrollment to follow-up, reported to be as low as 36% [6]. Technology can facilitate behavioral intervention research, but additional research is needed to understand the potential effects of technology-enabled approaches, such as electronic health records, patient portals, and clinical trials utilizing virtual visits, across the full course of participation, from the representativeness of reach and initial engagement to long-term retention. For instance, use of an electronic patient portal can extend participation opportunities to broader populations, yet might increase disparities in response rates [7]. These disparities highlight a key dilemma for researchers aiming to employ equitable yet scalable research trials, particularly in the tobacco and lung cancer screening fields characterized by low uptake, and even lower uptake among populations who would benefit most from these treatments and preventive care services [8, 9]. Identified barriers to using the patient portal include preference for in-person communication, no perceived need for the portal, lack of technical expertise with computers, privacy concerns, and no internet access [10].

Similarly, data are needed to understand whether genetic studies, particularly genomics-informed intervention trials, differentially engage participants across populations. Recent advances in precision health have led to genomics-informed behavior change interventions, in which polygenic risk scores and other personalized biomarker data guide optimal prevention and treatment strategies while motivating health behavior change [11]. Several studies, most notably involving tobacco and lung cancer risk, have found that patients respond favorably to receiving or discussing genetic risk information, including patients from populations who are not as represented in genetic research [12–15]. However, procedures involved in these genetic studies could impose unique barriers to study engagement, such as clinic visits for biospecimen collection, time delays associated with results processing, or the cognitive and emotional load of interpreting complex risk scores or results [16, 17]. These potential burdens raise new questions about individual-, contextual-, and temporal-specific characteristics of disengagement in these studies.

Some research has begun to address these complex knowledge gaps. A 2017 study analyzed sociodemographic factors as potential predictors of attrition for 1,147 potential participants with conditions suspected to have a genetic cause who were referred for an exome sequencing study. Race and distance to the enrollment site were significant predictors of enrollment, while race and education predicted retention at a 6-month follow-up [18]. Potential participants were approached via traditional outreach methods, by phone or in person during routine clinic visits. Furthermore, prior research [14–16] has acknowledged potential biases due to race and other sociodemographic factors in referral to genetic testing in primary care and other public health settings. Additionally, higher SVI may be associated with lower odds of undergoing breast, cervical, and colorectal cancer screening [19], lung cancer incidence and mortality [20], and lower odds of clinical trial participation [21]. Insurance coverage has also been found to improve the uptake of lung cancer screening [22, 23]. Therefore, it is important to understand factors related to engagement with more scalable recruitment technologies, as well as outside of individuals specifically referred for genetic testing.

The current study, situated in the context of two pragmatic trials aimed at the uptake of tobacco treatment and lung cancer screening, responds to and builds on prior work by leveraging modern technology-facilitated approaches to maximize potential for reach and representativeness across stages of (1) identification, (2) outreach, (3) enrollment, and (4) retention. We (1) identified a broad population as potentially eligible via electronic health records for one of two large-scale clinical trials and (2) advanced understanding of factors affecting engagement in response to newer participant recruitment tools such as the electronic patient portal. Attrition was examined across the full “cascade” of study activities, providing a more granular view of trajectories of engagement and retention through multiple clinical trials.

We examined two critical research questions: (1) To what extent do race, sociodemographic characteristics, and context-based factors predict study engagement? and (2) At which points along the trial continuum do these factors affect study engagement? Examining these questions in the context of genomics-informed behavioral interventions and modern study engagement technologies (e.g., electronic patient portal, virtual trial visits) has important public health implications. It may guide the design of more equitable and community-responsive strategies that improve representativeness, reduce disparities, and strengthen the population-level impact of clinical research.

## Methods

This study uses recruitment and participation data from two active large-scale cluster randomized clinical trials, the MOTIVATE and PRECISE trials, testing the impact of a multilevel precision treatment intervention aiming to address gaps in clinician and patient uptake of tobacco treatment (MOTIVATE) or lung cancer screening (PRECISE) in the primary care setting. The MOTIVATE Trial (NCT05846841), a personalized tobacco treatment study, is designed to test the impact of personalized tobacco treatment recommendations – guided by clinical, genetic, and nicotine metabolism biomarkers – on the uptake, adherence, and efficacy of tobacco treatment in primary care. In parallel, the PRECISE Trial (NCT05627674), a personalized prevention study, is designed to test the impact of patient-specific clinical and genetic risk feedback on primary care clinician and patient uptake of guideline-concordant lung cancer screening. The data analyzed for this study were acquired between March 2023 and May 2026.

### Study Population

Participants in the MOTIVATE Trial were eligible if they were an established patient in a participating clinic, aged 18 years or older, and smoked more than five cigarettes per day. Participants were excluded if they were currently using smoking cessation medication or had received a prescription for such medication within the past 30 days. Participants in the PRECISE Trial were eligible if they were an established patient of a participating primary care clinician, between the ages of 50 and 80, had a smoking pack-year history of more than 20 (e.g., 1 pack per day for 20 years, ½ pack per day for 40 years), had no history of lung cancer, had no prior lung cancer screening, and had no current order for lung cancer screening.

#### Setting

Both trials recruited participants from primary care clinics, including a diverse set of academic-affiliated and community-based clinics primarily in Eastern Missouri and Southern IL. The protocol for MOTIVATE was expanded in January 2026 to include specialty care clinics (e.g., cardiology, pulmonary, and stroke), although the vast majority of recruitment and enrollment has taken place within primary care; for PRECISE, all recruitment and enrollment has taken place within primary care. *Recruitment*: The recruitment approach did not differ meaningfully between the two trials. Patients were approached for study participation through phone calls, postal mail, and messages through their patient portal (when available). The trials used the same recruitment templates (e.g., flyer, mailed letter, patient portal message, and phone scripts), including highly similar advertising language about study compensation (USD 140 for MOTIVATE, USD 150 for PRECISE), free DNA and ancestry testing, and a personalized health report. Participants could indicate interest and initiate screening processes via phone, email, patient portal response, or a REDCap sign-up form linked to the study flyer. *Visit structure*: Both trials had a total of seven study visits that could be completed in-person, via videoconference (i.e., Zoom), or by phone. The patient decided on the modality for each study visit. In both trials, visit length ranged from approximately 15 min (follow-up assessments) to 1 h (baseline and genetic testing); of note, the time distances between follow-up assessments of 3-, 6-, and 12-months were greater than between preenrollment cascade steps (e.g., screening, consent, baseline assessment).

### Outcome Variables

Study engagement was analyzed across study recruitment and retention for each trial, modeling the “cascade” from initial outreach through retention at 3-,6-, and 12-months post-enrollment. This analysis was driven by a cascade of engagement framework, which was adopted from the cascade of care framework. The cascade of care framework was developed in response to the HIV/AIDS epidemic and has been applied across a wide range of diseases and care cascade frameworks for visualizing where gaps occur in health services so that interventions can be appropriately targeted for greatest impact [24].

#### Approached

Among primary care patients who were prescreened as eligible based on age and smoking status, research staff employed a combination of strategies to approach identified patients for study participation: mailed letters, phone calls, and, when available, electronic messages sent through the patient portal.

#### Reached

Patients who responded to the initial approach in any way (indicating interest, declining interest, or completing eligibility screening) were considered to be reached.

#### Screened for Eligibility, Determined Eligible, and Consented

When reached, interested patients completed a telephone screening to determine study eligibility. Eligible patients who wished to participate were then asked to consent to the study. Although eligibility status does not reflect a specific behavior within the cascade, it remains an important part of the enrollment cascade by allowing for a cleaner analysis of subsequent steps in the cascade (e.g., consent) while maintaining a consistent flow of sample size attrition. In addition, analyzing eligibility status provides an opportunity to examine representativeness of the eligibility criteria, which is especially relevant given that black patients may be less likely to meet eligibility criteria for lung cancer screening than white patients, despite having similar risk of developing lung cancer [25]. With US Preventive Services Task Force guidelines for lung cancer screening being updated in 2021 and this study being conducted between 2023 and 2026, analysis of this step presents a timely opportunity to determine whether potential racial and other inequities in eligibility may have been mitigated in the wake of guideline changes.

Following consent, patients completed a preenrollment study visit (conducted in-person or remotely via Zoom or phone) which included a baseline assessment and completion of biological (saliva nicotine metabolism rate) and genetic testing. When conducted remotely, kits were mailed to participants who were then guided through the completion process by study staff.

#### Completed Genetic Testing and Enrolled

Completion of genetic testing was a proxy for enrollment, when the patient and clinician received the behavior change intervention materials. This timepoint interfaces with several potential barriers unique to genetic studies that may affect engagement at this step; these include trust and comprehension of risks and benefits of these studies, influencing the decision to donate genetic samples, the willingness to wait for genetic results, and potential technological hurdles of transferring personal genetic data to the study portal. Of note, patients acknowledged during the consent process that if randomized into a group without genetic risk feedback, they would receive this information at the end of the trial.

#### Retained Through Follow-Up

Post-enrollment study visits included 3-, 6-, and 12-month follow-up assessments. These specific follow-up assessments were used because they were consistent between the two trials (MOTIVATE has a 1-month follow-up and PRECISE has an 18-month follow-up that were excluded from analyses). A patient was considered to have entered the follow-up period if they had been in the trial long enough to complete at least the 3-month period. Patients were considered lost to follow-up if there was an extended period of inactivity from when they completed their latest step to the date of data acquisition for this analysis; see Table 1 for the windows of inactivity and follow-up timeframes used.

**Table 1.** Definition of outcomes across the cascade of study participation

Term	Definition	Required timeframe for completion relative to prior step
Approached	Patient was sent an invitation via one or more methods to learn about the study from a research staff member after being identified as prescreen eligible through their health record	N/A
Reached	Research staff member received any response from the patient (e.g., answering or returning phone call, expressing interest or declining to participate in patient portal)	2 Months
Screened for eligibility	Patient completed eligibility screening for MOTIVATE or PRECISE trials	2 Months
Eligible to participate	Patient met inclusion criteria for the respective trial to which they were screened	N/A
Consented	Patient completed the consent process, formalizing their agreement to participate in the trial	1 Month
Completed genetic testing	Patient completed the genetic testing component, including saliva sample donation and genetic data sharing with study team	3 Months
Enrollment	*(Not an Outcome)* Procedural step for randomization and intervention delivery	N/A
Retained through follow-up	Patient completed the last eligible follow-up visit (3-, 6-, or 12-month), based on their current window at the time of data acquisition	3-Month: 1.5–4.5 mo 6-Month: 4.5–9 mo 12-Month: 9–15 mo

### Outcome Variable Approach

The binary outcome of each step in the cascade of study participation was whether a patient completed each step. Table 1 includes definitions for each outcome. Patient milestones and engagement were tracked by research staff and stored in REDCap databases [26, 27]. Data from both trials were combined into one dataset for primary analyses to identify general trends in study engagement. Secondary analyses were conducted to identify factors associated with engagement stratified across each trial. These stratified analyses were performed to account for the differences in trial focus, eligibility criteria, age distribution, and the types of clinics from which patients were recruited, and to examine overlapping and distinct patterns.

### Predictor Variables Associated with Study Engagement

For all approached patients, their age, race, gender, and primary insurance coverage status were obtained as standard fields in the Epic electronic health record. Due to very small proportions of patients who did not identify as black or white, analyses were limited to those two categories (94.6% of patients were either black or white). Primary insurance coverage status denotes whether a patient’s coverage was able to be successfully confirmed with the payer and is categorized as either “verified” or “not verified”. Due to differences in the age requirements between the two trials, age was not included in the primary analysis. In the stratified analyses, age was categorized as “18–34,” “35–49,” “50–64,” “65–80,” and “>81”.

A social vulnerability index (SVI) score was determined for each patient based on geocoded census tract information using the recruitment address information from the trial databases. Commonly used in public health and health equity research as a measure of community-level need, SVI is a place-based index derived from 16 US Census variables grouped into four major areas of social vulnerability: socioeconomic status, household characteristics, racial and ethnic minority status, and housing type and transportation. An index is derived for each of these themes and then combined into a single measure of overall social vulnerability [28]. As in prior research, SVI scores were classified into two groups, which were categorized as either “lower” (SVI score of <0.5, 51.4% of patients) or “higher” (SVI score of >0.5, 43.9% of patients) [29–31].

The distance from a patient’s residence to the study site was calculated using the Haversine formula and was categorized into “closer” or “farther” based on a median split (10.61 mi) [32, 33]. “Closer” distances represented a proxy for urban residence as the study site was in an urban area, and “farther” distances represented a proxy for rural and other nonmetropolitan residences.

### Analyses

Descriptive statistics were used to characterize each covariate and its distribution. For each step in the cascade of participation, study engagement was modeled using univariate modified Poisson regression [34, 35] models for the previously defined covariates. The outcome of each analysis was binary: whether the patient completed or did not complete the current step. Risk ratios (RRs) were reported with 95% confidence intervals (CIs) and *p* values. Significant predictors from the univariate models were then included in multivariate analyses. Insurance status was only analyzed in univariate analyses due to the large amount of missing data (70.9%). Analyses were conducted at a significance threshold of *p* < 0.05 with corrections for multiple comparisons using false discovery rate corrections at each step of the cascade [36]. All analyses were completed using R version 4.3.2 [37].

Using the same analytic parameters, a secondary analysis was performed among patients who had an active patient portal (i.e., opened account with enabled communication about research participation opportunities) to analyze factors of study engagement for patients who were recruited through their electronic patient portal in addition to more traditional outreach methods (phone calls and postal mailings). This analysis was performed to evaluate whether any additive benefit of patient portal recruitment existed for outcomes of reach and representativeness. In addition to comparing patient portal versus non-patient portal recruitment, we further distinguished with analyses between patients reached directly through the patient portal versus those who were sent a patient portal message but were ultimately reached (i.e., directly contacted) through other methods, most commonly by phone. This was considered to be reached indirectly outside of the portal as, conceivably, the patient portal messages may increase the likelihood of reaching individuals by phone (e.g., via increased study name recognition, or prior intentions to respond). In addition, stratified analyses for MOTIVATE and PRECISE were computed for trial-specific results.

Finally, exploratory analyses were performed to determine any potential effect of receiving genetic risk and the level of genetic risk (i.e., for developing lung cancer) on retention to follow-up. While all participants completed genetic testing, this analysis compared participants who were randomized to either receive genetic risk results or not (i.e., usual care [UC] with receipt of genetic risk results following completion of the study). First, participants who received genetic risk results were compared with UC (Gen vs. UC), and each level of genetic risk result from lowest to highest (Gen-lowest, Gen-middle, Gen-highest) was compared with UC as the reference using the same analytic parameters. Pairwise comparisons were also calculated between each of the genetic risk levels.

## Results

### Variability in Outcomes of Study Engagement

Across trials, a total of 24,366 patients were approached. Table 2 includes descriptive statistics of all patients who were approached for either trial. Of those approached, 10,686 patients were reached (43.9%). Attrition across individual steps of the cascade ranged from 38.4% (Screened → Eligible) to 83.7% (Genetic Testing Completed → Retained Through Follow-Up). Figure 1 presents the cascade of study engagement across all trial engagement outcomes.

**Table 2.** Sociodemographic and trial engagement characteristics

Term	Overall (*N* = 24,366)
Reached	
Yes	10,686 (43.9%)
No	12,086 (49.6%)
Other/Missing	1,594 (6.5%)
Screened	
Yes	4,511 (18.5%)
No	16,728 (68.7%)
Other/Missing^a^	3,127 (12.8%)
Eligibility Status	
Yes	1,734 (7.1%)
No	19,505 (80.1%)
Other/Missing	3,127 (12.8%)
Consented	
Yes	1,119 (4.6%)
No	21,197 (87.0%)
Other/Missing	2,050 (8.4%)
Completed Genetic Testing	
Yes	729 (3.0%)
No	21,579 (88.6%)
Other/Missing	2,058 (8.4%)
Entered follow-up timeframe	
Yes	662 (2.7%)
No	117 (0.5%)
Other/Missing	23,587 (96.8%)
Retained through follow-up	
Yes	554 (2.3%)
No	21,629 (88.8%)
Other/Missing	2,183 (9.0%)
Age	
18–39	3,386 (13.9%)
40–59	8,549 (35.1%)
60–79	11,649 (47.8%)
80–99	700 (2.9%)
Other/Missing	82 (0.3%)
Gender^b^	
Male	12,227 (50.2%)
Female	12,082 (49.6%)
Other/Missing	57 (0.2%)
Race^c^	
Black or African American	6,726 (27.6%)
White	16,578 (68.^c^0%)
Other/Missing	1,062 (4.4%)
Insurance Coverage	
Not Verified	2,992 (12.3%)
Verified	3,254 (13.4%)
Other/Missing	18,120 (74.4%)
Distance from study site	
Closer	12,185 (50.0%)
Farther	12,181 (50.0%)
SVI	
Lower	15,678 (64.3%)
Higher	8,688 (35.7%)

^a^ Other/Missing: Data that were not analyzed due to not being present in the dataset or due to small sample sizes. For steps in the cascade, the missing data is for all approached patients; however, the sample for each analysis was filtered to the sample that completed the previous step in the cascade.

b We conducted additional analyses and were reassured to find that, of the 1,034 patients that provided self-reported gender information at baseline, 1,034 matched the gender information in the EMR.

c Of the 1,014 patients that provided self-reported race information at baseline, 1,013 (99.9%) matched the race information in the EMR.

**Fig. 1.**
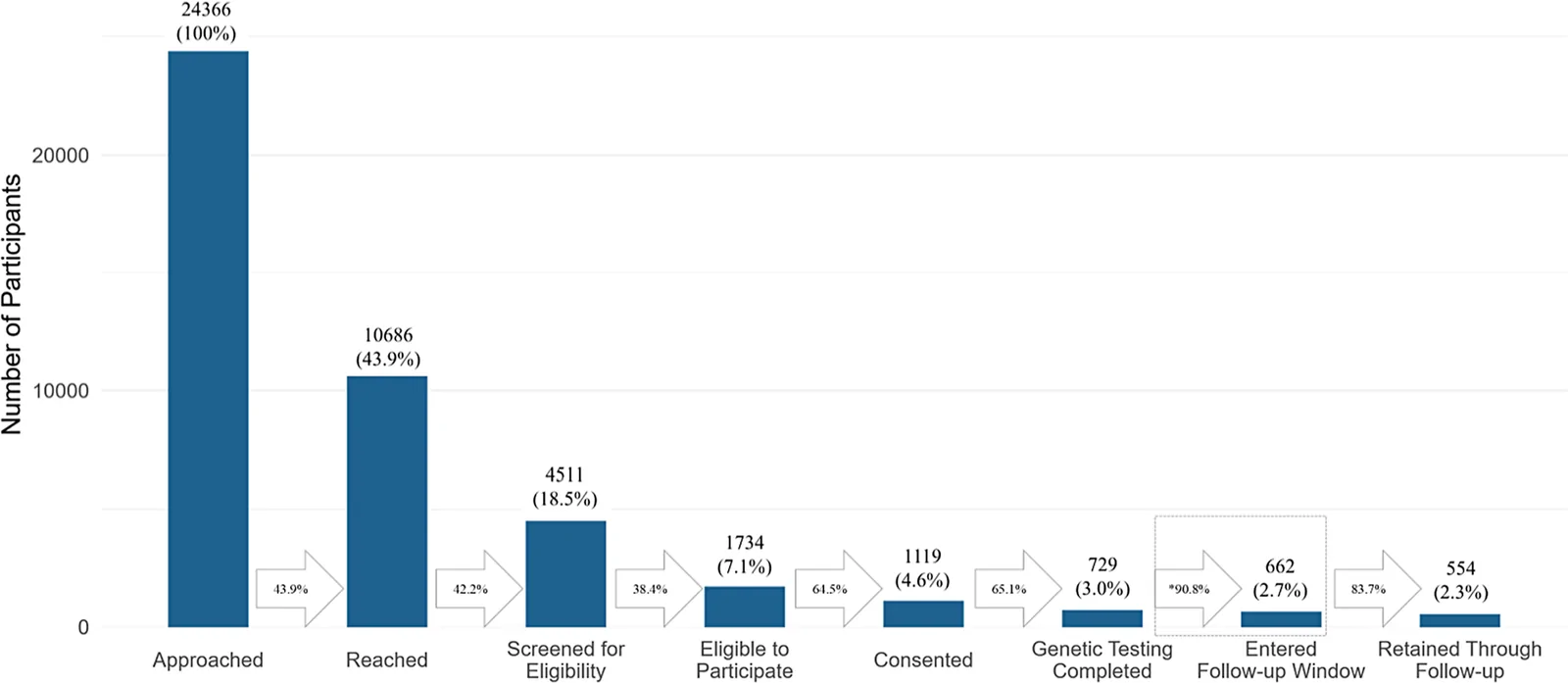
Number of participants at each step of the cascade of participation. *Participants enrolled in the study long enough to be eligible for the first follow-up. Not an analyzed outcome but serves as the denominator for retained through follow-up.

### Predictors of Study Engagement

#### Reached

Univariate analyses showed that black patients were more likely to be reached compared to white patients (RR: 1.04; 95% CI: 1.01–1.07; *p* = 0.022), patients who resided farther from the study site were more likely to be reached compared to those who resided closer (RR: 1.06; 95% CI: 1.03–1.09; *p* < 0.001), patients who resided in areas with higher social vulnerability were more likely to be reached (RR: 1.06; 95% CI: 1.03–1.09; *p* < 0.001), and patients with verified insurance coverage were more likely to be reached than those without verified insurance (RR: 1.28; 95% CI: 1.20–1.38; *p* < 0.001). See online supplementary Table 1 (for all online suppl. material, see https://doi.org/10.1159/phg/aenag004) for results from the univariate analyses. In multivariate analyses, black patients remained more likely to be reached (RR: 1.07; 95% CI: 1.04–1.11; *p* < 0.001), patients who lived farther remained more likely to be reached (RR: 1.1; 95% CI: 1.07–1.13; *p* < 0.001), and patients from higher SVI areas remained more likely to be reached (RR: 1.07; 95% CI: 1.04–1.1; *p* < 0.001). See online supplementary Table 2 for results from the multivariate analyses and Figure 2 for a visualization of significant covariates from univariate and multivariate analyses.

**Fig. 2.**
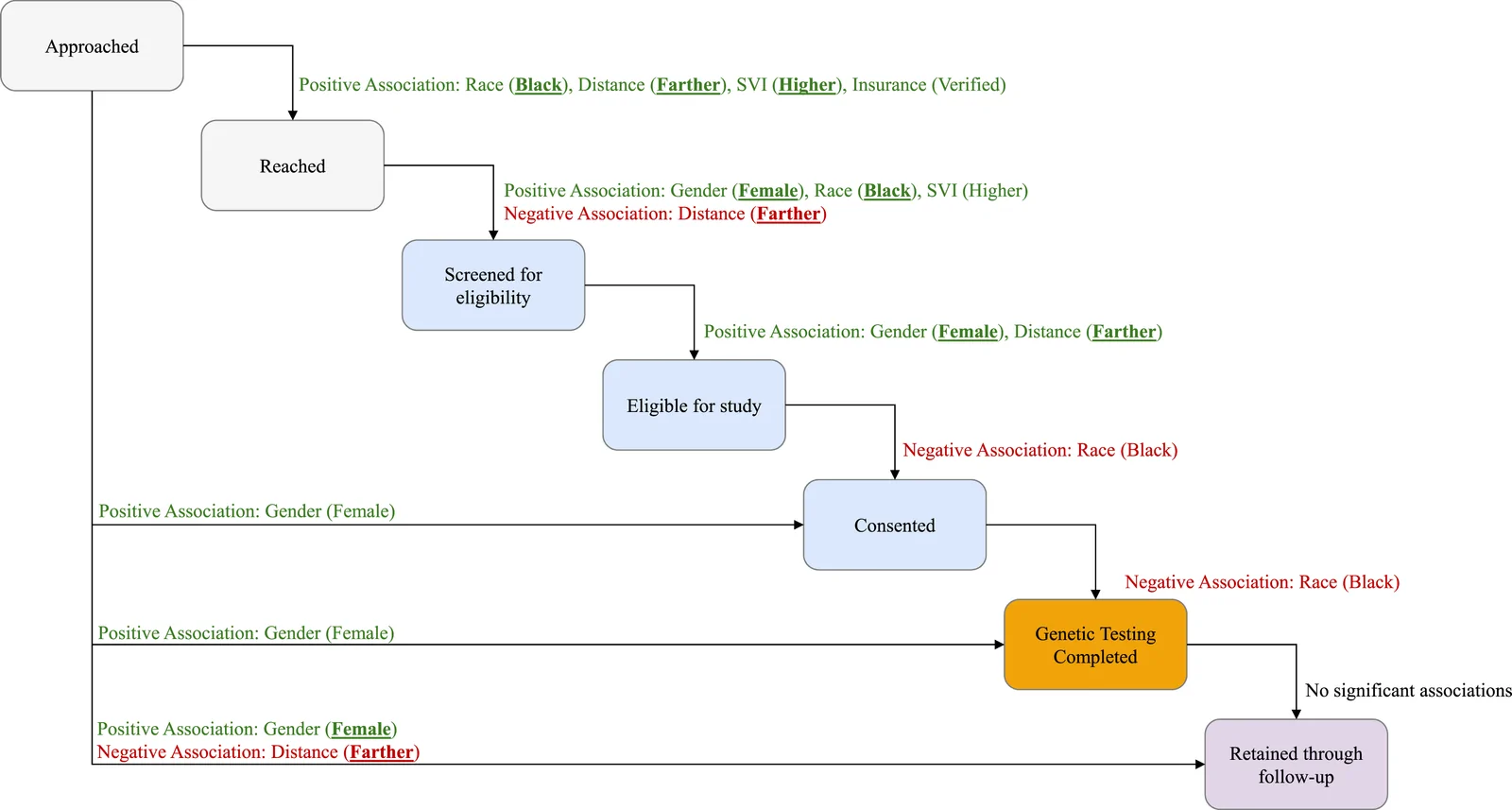
Factors associated with patient engagement. (i) Gender (ref.: male), (ii) race (ref.: white), (iii) distance (ref.: closer), (iv) SVI (ref.: lower), (v) age (ref.: 50–64 years), (vi) insurance (ref.: not verified), and (vii) group denotes significance in multivariate model.

#### Screened

Of the 10,686 patients reached, 4,511 (42.2%) completed eligibility screening. Univariate analyses showed that female patients were more likely to complete eligibility screening than male patients (RR: 1.07; 95% CI: 1.02–1.12; *p* = 0.004). Black patients were more likely to complete eligibility screening than white patients (RR: 1.38; 95% CI: 1.32–1.44; *p* < 0.001). Patients who lived farther from the study site were less likely to complete eligibility screening (RR: 0.72; 95% CI: 0.69–0.76; *p* < 0.001). Patients who resided in areas with higher SVI were more likely to complete eligibility screening (RR: 1.06; 95% CI: 1.01–1.11; *p* = 0.020). These associations remained significant in multivariate analyses for Female patients (RR: 1.06; 95% CI: 1.02–1.11; *p* = 0.009), black patients (RR: 1.24; 95% CI: 1.17–1.30; *p* < 0.001) and for patients living farther from the study site (RR: 0.79; 95% CI: 0.75–0.84; *p* < 0.001).

#### Eligible

Of the 4,511 patients who completed eligibility screening, 1,734 (38.4%) were found to be eligible. Univariate analyses showed that female patients were more likely to be deemed eligible compared to male patients (RR: 1.18; 95% CI: 1.09–1.27; *p* < 0.001). Patients who resided farther from the study site were more likely to be deemed eligible compared to those who lived closer (RR: 1.11; 95% CI: 1.03–1.2; *p* = 0.010). These associations remained significant in multivariate analyses (RR: 1.18; 95% CI: 1.09–1.27; *p* < 0.001) for female patients and (RR: 1.12; 95% CI: 1.04–1.21; *p* = 0.003) for patients who lived farther from the study site.

#### Consented

Of the 1,734 patients who were eligible for study inclusion, 1,119 (64.5%) ultimately consented to participate in the respective trial featuring a genomics-informed behavior change intervention. Contrary to early steps in the cascade, univariate analyses showed that black patients were less likely to consent than white patients (RR: 0.81; 95% CI: 0.75–0.88; *p* < 0.001). No multivariate analyses were performed for the consented outcome due to only one variable in univariate analyses (i.e., race) having a significant association.

#### Completed Genetic Testing

Of the 1,119 patients who consented, 729 (65.1%) completed genetic testing and thus were queued for enrollment and behavioral intervention. Contrary to early steps in the cascade, univariate analyses showed that black patients were less likely to complete genetic testing compared to white patients (RR: 0.84; 95% CI: 0.75–0.93; *p* = 0.003). No multivariate analyses were performed for the completed genetic testing outcome due to only one variable in univariate analyses (i.e., race) having a significant association.

#### Retained Through Follow-Up

Of note, no significant associations were found in univariate or multivariate analyses for the outcome of being retained through follow-up among those who had completed genetic testing.

#### Longer-Range Associations

Although no multivariate associations were found for the consented outcome in the step-by-step analyses, gender predicted the likelihood of consent among patients who were approached for study participation. For patients who were approached, female patients were more likely to consent to study participation (RR: 1.28; 95% CI: 1.14–1.44; *p* < 0.001), complete genetic testing (RR: 1.28; 95% CI: 1.11–1.48; *p* = 0.003), and be retained through follow-up (RR: 1.32; 95% CI: 1.12–1.56; *p* = 0.004) than male patients. Approached patients who resided farther from the study site were less likely to be retained through follow-up (RR: 0.82; 95% CI: 0.7–0.97; 0.04). See Figure 2 for a visualization of the longer-range associations.

#### Trial-Specific Results

For MOTIVATE, we find a similar reversal of association in engagement for black patients to the primary analyses, where black patients are more likely to be reached (RR: 1.16; 95% CI: 1.11–1.21; *p* < 0.001), screened (RR: 1.26; 95% CI: 1.19–1.33; *p* < 0.001), and be deemed eligible (RR: 1.17; 95% CI: 1.06–1.29; *p* = 0.006) in the trial, but less likely to provide consent to participate in the trial (RR: 0.81; 95% CI: 0.73–0.91; *p* = 0.001) compared to white patients. Conversely, for PRECISE, race was negatively associated throughout the cascade, except for being screened, where black patients were more likely to complete eligibility screening compared to white patients (RR: 1.56; 95% CI: 1.45–1.69; *p* < 0.001). Black patients were less likely to be reached (RR: 0.93; 95% CI: 0.89–0.97; *p* = 0.002), be eligible for trial participation (RR: 0.71; 95% CI: 0.62–0.81; *p* < 0.001), provide consent (RR: 0.83; 95% CI: 0.74–0.93; *p* = 0.005), and complete genetic testing (RR: 0.71; 95% CI: 0.59–0.86; *p* = 0.002). Patients who resided farther from the study site were less likely to complete eligibility screening in both MOTIVATE (RR: 0.8, 95% CI: 0.76–0.85; *p* < 0.001) and PRECISE (RR: 0.68; 95% CI: 0.63–0.73; *p* < 0.001). However, residing farther from the study site was positively associated with being reached (RR: 1.05; 95% CI: 1.01–1.09; *p* = 0.046) and being eligible (RR: 1.31; 95% CI: 1.16–1.47; *p* < 0.001) only in PRECISE. Patients who resided in higher SVI areas were more likely to be screened in both MOTIVATE (RR: 1.14, 95% CI: 1.08–1.21; *p* < 0.001) and PRECISE (RR: 1.15; 95% CI: 1.07–1.24; *p* = 0.001). In MOTIVATE, female patients were more likely to be eligible (RR: 1.35; 95% CI: 1.22–1.5; *p* < 0.001), whereas in PRECISE, female patients were more likely to be reached (RR:1.08; 95% CI: 1.04–1.12; *p* < 0.001) and screened (RR: 1.14; 95% CI: 1.05–1.23; *p* = 0.004). Having verified insurance was positively associated with being reached in both MOTIVATE (RR: 1.42; 95% CI: 1.28–1.57; *p* < 0.001) and PRECISE (RR: 1.13; 95% CI: 1.03–1.24; *p* = 0.011). However, having verified insurance was negatively associated with being screened for MOTIVATE only (RR: 0.89; 95% CI: 0.79–0.99; *p* = 0.035).

For within trial age analyses, age was significantly associated in varying ways throughout the cascade for MOTIVATE (reached, screened, eligible, and completing genetic testing) and for longer-range associations (approached to reached, approached to completing genetic testing, approached to retained through follow-up). In PRECISE, age was significantly associated in varying directions with being reached and screened. It is difficult to compare the role of age across trials given the different age distributions between trials, with MOTIVATE having wider age ranges (18 years and above) compared to PRECISE (50–80 years). The within-trial analyses can be found in online supplementary Table 3 and online supplementary Figure 1 (MOTIVATE) and online supplementary Table 4 and online supplementary Figure 2 (PRECISE).

### Electronic Patient Portal Analyses

Of the 24,366 total patients who were approached, 18,154 had patient portal accounts enabled to receive communication about research participation and were approached for study participation through the portal, in addition to the traditional outreach methods. Of these patients, 2,511 (13.8%) were directly reached through the portal, with 859 (34.2%) indicating interest in the studies and 1,552 (65.8%) declining interest. See Figure 3 for a visualization of patients who were approached via the patient portal.

**Fig. 3.**
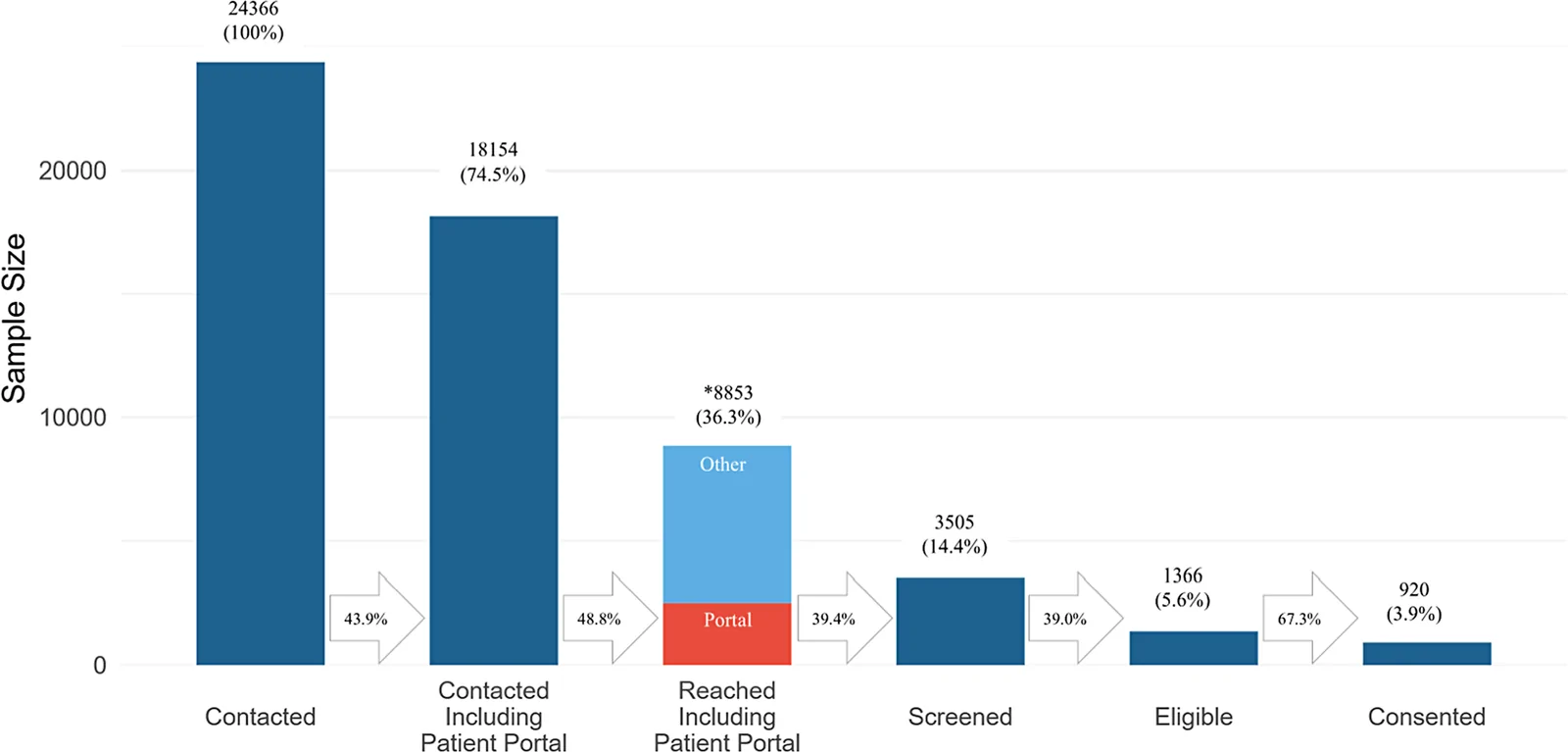
Number of participants contacted through the patient portal at each step of the cascade of participation. *8,853. (i) Of 8,853 participants, 2,511 responded in the patient portal (859 indicated interest) and (ii) 6,342 responded to other outreach methods.

#### Approached via Portal

In univariate analyses for patients who were approached for study participation through their research communication-enabled portals, female patients were more likely to be approached through the portal (RR: 1.09; 95% CI: 1.07–1.10; *p* < 0.001) compared to male patients. Black patients were less likely to be approached through the portal (RR: 0.72; 95% CI: 0.70–0.73; *p* < 0.001) than white patients. Patients from higher SVI areas were less likely to be approached through the electronic patient portal (RR: 0.88; 95% CI: 0.87–0.9; *p* < 0.001) than those from lower SVI areas. Patients who lived farther from the study site were more likely to be approached through the patient portal (RR: 1.21; 95% CI: 1.19–1.23; *p* < 0.001). Patients with verified insurance were more likely to be approached through the patient portal (RR: 1.13; 95% CI: 1.09–1.18; *p* < 0.001). In multivariate analyses, female patients remained more likely to be approached via the portal (RR: 1.1; 95% CI: 1.08–1.11; *p* < 0.001), black patients remained less likely to be approached through the portal (RR: 0.75; 95% CI: 0.74–0.77; *p* < 0.001), patients who lived farther remained more likely to be approached through the portal (RR: 1.08; 95% CI: 1.07–1.10; *p* < 0.001), and patients from higher SVI areas remained less likely to be approached through the portal (RR: 0.93; 95% CI: 0.91–0.94; *p* < 0.001). See Figure 4 for a visualization of significant predictors of study engagement for patients approached via the patient portal.

**Fig. 4.**
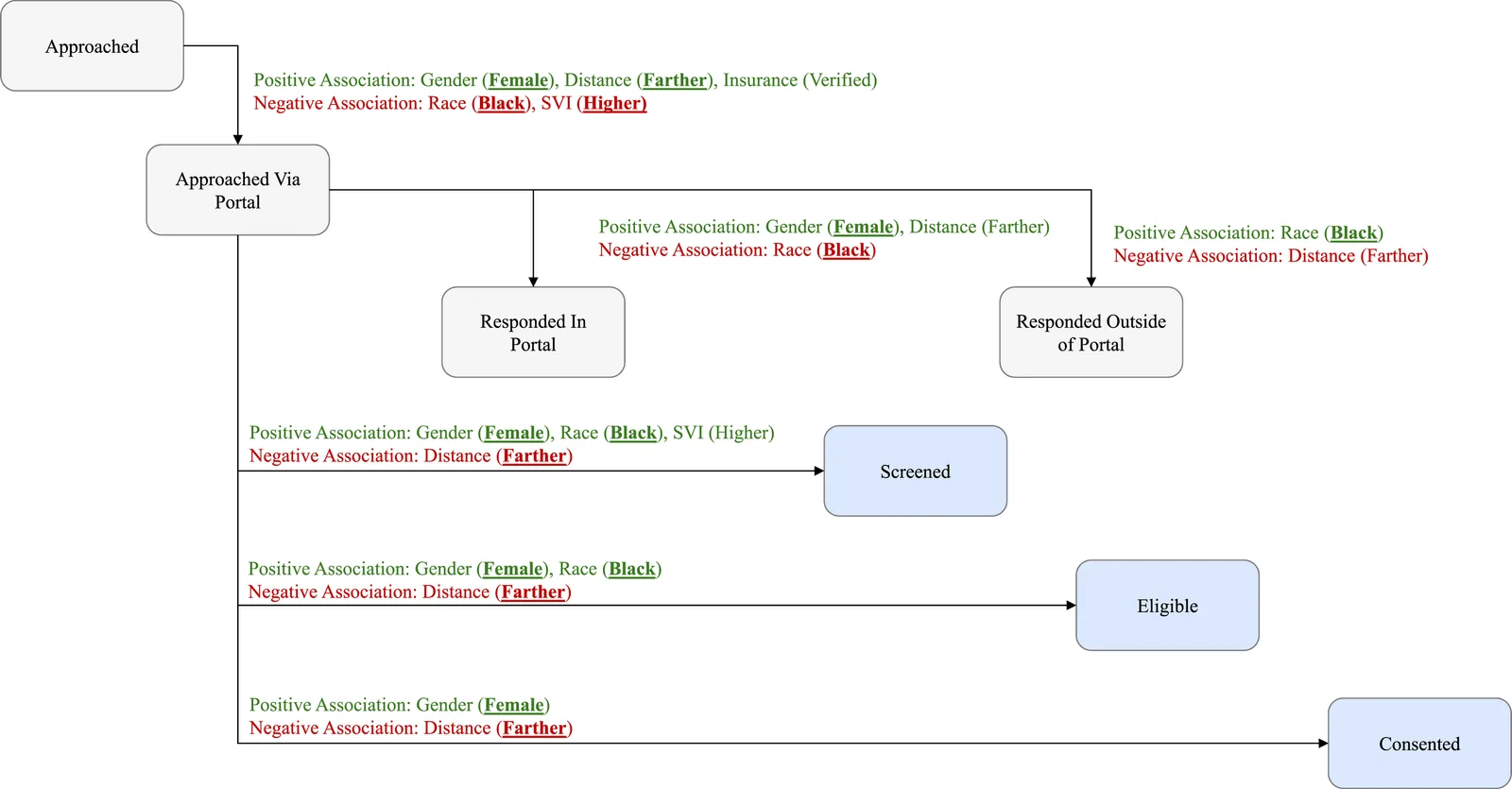
Factors associated with patient engagement for patients contacted via the patient portal. (i) Gender (ref.: male), (ii) race (ref.: white), (iii) distance (ref.: closer), (iv) SVI (ref.: lower), (v) age (ref.: 50–64 years), (vi) insurance (ref.: not verified), and (vii) group denotes significance in multivariate model.

#### Reached: Directly in Portal

Patients who explicitly selected either “Interested” or “Decline” in their portal message were considered to have been reached directly in the portal. Of the 18,154 patients who were approached via the patient portal, female patients were more likely to respond directly in the patient portal (RR: 1.09; 95% CI: 1.02–1.18; *p* = 0.021), black patients were less likely to respond in the patient portal (RR: 0.56; 95% CI: 0.51–0.63; *p* < 0.001), patients who lived farther away were more likely to respond in the portal (RR: 1.19; 95% CI: 1.11–1.28; *p* < 0.001). Several covariates remained significant after multivariate analyses (RR: 1.12; 95% CI: 1.04–1.2; *p* = 0.006) for female patients and (RR: 0.57; 95% CI:0.51–0.63; *p* < 0.001) for black patients.

#### Reached: Indirectly Outside of Portal

Patients who did not respond to the patient portal message that was sent, but were still reached through other methods (mail or phone call) were considered to have been reached indirectly outside of the portal. Black patients were more likely to be reached outside of the patient portal (RR: 1.29; 95% CI: 1.23–1.34; *p* < 0.001), and patients living farther away were less likely to be reached outside of the patient portal (RR: 0.94; 95% CI: 0.91–0.98; *p* = 0.008). In multivariate analyses, black patients remained more likely to be reached outside of the portal (RR: 1.3; 95% CI: 1.24–1.36; *p* < 0.001).

#### Screened

Of the patients who were approached via the patient portal, Female patients were more likely to complete eligibility screening (RR: 1.09; 95% CI: 1.02–1.15; *p* = 0.006). Black patients were more likely to complete eligibility screening (RR: 1.48; 95% CI: 1.39–1.58; *p* < 0.001). Patients who lived farther were less likely to complete eligibility screening (RR: 0.74; 95% CI: 0.70–0.78; *p* < 0.001). Patients from higher SVI areas were more likely to complete eligibility screening (RR: 1.09; 95% CI: 1.03–1.16; *p* = 0.006). In multivariate analyses, Female patients remained more likely to complete eligibility screening (RR: 1.08; 95% CI: 1.02–1.15; *p* = 0.017), black patients remained more likely to complete eligibility screening (RR: 1.35; 95% CI: 1.26–1.45; *p* < 0.001), and patients who lived farther remained less likely to complete eligibility screening (RR: 0.82; 95% CI: 0.77–0.88; *p* < 0.001).

#### Eligible

Of the patients who were approached via the patient portal, female patients were more likely to be eligible for participation compared to male patients (RR: 1.28; 95% CI: 1.15–1.42; *p* < 0.001), black patients were more likely to be eligible for participation (RR: 1.39; 95% CI: 1.24–1.55; *p* < 0.001), and patients who resided farther away were less likely to be eligible (RR: 0.84; 95% CI: 0.76–0.93; *p* = 0.001). Gender and race remained significant in multivariate analyses (RR: 1.26; 95% CI: 1.13–1.44; *p* < 0.001) for female patients and (RR: 1.32; 95% CI: 1.17–1.48; *p* < 0.001) for black patients.

#### Consented

Of the patients who were approached via the patient portal, female patients were more likely to consent compared to male patients (RR: 1.27; 95% CI: 1.12–1.44; *p* < 0.001) and patients who lived farther were less likely to consent (RR: 0.85; 95% CI: 0.75–0.97; *p* = 0.026). These predictors remained significant in multivariate analyses for female patients (RR: 1.26; 95% CI: 1.11–1.44; *p* < 0.001) and for patients residing farther away (RR: 0.86; 95% CI: 0.76–0.98; *p* = 0.021).

### Genetic Risk Analyses

No significant differences in retention to follow-up were found for patients who received genetic risk results compared to the UC group who did not receive any genetic risk results (Gen vs. UC). Additionally, no significant pairwise associations were found between risk levels (Gen-lowest, Gen-middle, Gen-highest) for patients who received genetic risk results. See online supplementary Table 5 for detailed results of these analyses.

## Discussion

In this analysis of more than 24,000 patients across two active pragmatic clinical trials testing the impact of genomic-informed behavior change interventions, we identified distinct, stage-specific patterns of study engagement across the cascade of activities ranging from initial patient outreach to retention through follow-up visits. To our knowledge, this is the first study to detect a reversal of associations between race and study engagement within the cascade, most notably with black patients having higher interest in study participation but lower engagement later in consent and study procedures. There was also a surprising change in factors influencing study engagement through the patient portal, with patients living more distally having higher engagement early but lower engagement later. This corresponds with some prior research which found that patients who lived farther from the study site were less likely to enroll in clinical genomics research, perhaps as a proxy for disparities in rural-residing individuals [18, 38, 39]. However, findings are inconsistent with some studies which found that patients who were black and lived in more vulnerable areas had lower trial engagement overall [21, 40–42]. Those studies might not have followed participants across the cascade of study engagement.

While existing multimodal outreach strategies may be effective in reaching underrepresented populations including black patients and those living in areas with a higher SVI, challenges persisted in engaging black patients downstream in the cascade (i.e., consent, completing genetic testing). There may be systemic and structural factors or a reluctance to trust research activities in the context of historical injustices particularly in genetic research [43–45]. Findings highlight that engagement is not a single event, but a dynamic process, with different sociodemographic, contextual factors, and trial logistics influencing participation at each stage. Future research could investigate mechanisms underlying the complex processes and influences that appear to shape participation patterns at various stages of the engagement cascade.

Importantly, patterns of engagement also differed by outreach modality. Female patients, patients with verified insurance, and those residing farther from the study site were more likely to be approached through the electronic patient portal, whereas black patients and those living in areas with higher SVI scores were less likely to be approached through the portal. Among patients approached via the portal, black patients were less likely to respond electronically via the portal but were more likely to be reached through alternative methods such as mail or telephone. In contrast, patients residing farther from the study site were more likely to respond through the portal and less likely to be reached through nonelectronic patient portal methods. Together, these findings suggest that combining these methods is nonredundant and may reduce inequities in reach due to differences in which populations have access to and utilize their patient portal. To our knowledge, this is also the first study to detect this reversal of associations for race- and place-based predictors of trial engagement as a function of electronic versus nonelectronic recruitment methods. These findings highlight the limitations of relying on a single outreach modality and underscore the importance of maintaining multimodal recruitment strategies to promote equitable engagement and representativeness.

Interestingly, this study did not find that receiving genetic risk scores, or the level of reported genetic risk of developing lung cancer, influenced patient disengagement at follow-up; retention remained high across study arms and levels of genetic risk results. This corresponds with prior research showing that individuals generally do not perceive decision regret following receipt of lung cancer-related genetic risk information [15, 46]. At the same time, those who were randomized to not receive genetic results until study completion had similarly high retention, perhaps corresponding with previous findings showing high interest and enthusiasm among individuals who smoke for receiving genetic risk results related to lung cancer [13, 47].

A key strength of this study is its granular examination of engagement across multiple stages of the participation cascade in two large-scale clinical trials. This approach, combined with a larger sample than in prior research, revealed nuanced patterns that would not be apparent using traditional binary measures of participation, including the observed shift in engagement among black patients from early to later trial stages. The persistent challenges to secure and sustain participation and engagement among populations historically underrepresented in research point to the need for targeted, stage-specific engagement and retention strategies. On more of a policy level, we found evidence that black patients in this sample were less likely to be eligible for the PRECISE trial, which aligned with current lung cancer screening guidelines. This may reflect a persistent challenge to remove inequities in lung cancer screening eligibility even in the wake of updated guidelines that, in part, intended to address this issue. Additionally, our analysis of outreach modality provides actionable insight into how different populations respond to digital versus non-digital recruitment methods, which is increasingly relevant as trials expand the use of patient portals and other electronic tools. This study contributes novel evidence to guide the development and deployment of engagement strategies that are responsive to timing and mode-specific challenges to participation in genomics-informed intervention trials.

This study does have some key limitations. Other individual-level factors such as income, educational attainment, access to technology, and employment status were not available and may have influenced engagement patterns. It should be noted that since both trials recruited for study participation through primary care providers, the effect of a patient having an assigned primary care provider on trial engagement could not be considered in this study. Similarly, recruited patients were not asked about the type of clinic in which they were seen, so clinical setting (e.g., community or academic) and other clinic-level variables could not be included in this study. Future work could examine differential enrollment or engagement based on having a regular or primary provider, or by type of clinical setting. We also acknowledge that the extent of use of the patient portal, rather than the availability of the patient portal, could have helped to refine the analyses, but these data were not available. Both trials were based in the central Midwest and remain ongoing, and although overall patterns are likely stable given the large sample of more than 24,000 patients, participant statuses may shift as retention efforts continue and could have differed in other geographic regions. Finally, while these analyses identify associations between participant characteristics and engagement, they do not elucidate the underlying mechanisms driving these patterns. Future work could look at the associations between baseline variables, such as perceived benefit about the intervention and attitudes towards genetic testing, and retention through follow-up and the uptake of lung cancer screening and tobacco treatment. The integrated use of qualitative and community-engaged approaches may serve to better understand the social, structural, and contextual factors shaping engagement and to inform interventions that support equitable participation throughout the full cascade of study participation.

Findings from this work, combined with advances in electronic health records, clinical trial engagement metrics, and machine learning techniques, may present future opportunities to move beyond retrospective analyses toward integrated, predictive models that can proactively identify participants less likely to engage in study activities. Such approaches can support more responsive and equitable implementation strategies, particularly when paired with community-informed perspectives that account for social context, trust, and structural barriers to participation.

## Conclusion

This study found that study engagement in behavioral and preventive health trials varies dynamically across multiple stages of the participation cascade and varies systematically by outreach modality and race- and place-based sociodemographic characteristics. Our findings highlight that initial successes with engaging historically underrepresented populations, particularly black patients, may not necessarily translate into sustained participation, underscoring the need for stage-specific, equity-centered engagement strategies. By characterizing engagement patterns across large-scale, real-world trials, this work provides a foundation for more adaptive, responsive, and equitable trial designs, including just-in-time interventions that may improve representativeness, enhance validity, and support the reach and uptake of evidence-based interventions to the populations that need them the most.

## Acknowledgments

The authors gratefully acknowledge Jaeheon Kim, Genevieve Slavens, Vidhya Venkataraman, Myranda Vitale, Aaron Villamayor, Mohamed Abdulsattar, and Joshua Clark for their assistance with data collection and for their thoughtful feedback, which contributed significantly to strengthening the quality, clarity, and presentation of this work.

## Statement of Ethics

This study protocol was reviewed and approved by an Institutional Review Board in the Human Research Protection Office at Washington University in St. Louis (St. Louis, MO, IRB ID: 202502015). All procedures were conducted in accordance with the World Medical Association Declaration of Helsinki. As determined by the Human Research Protection Office at Washington University in St. Louis, written informed consent was not required, and this project was granted a waiver of HIPAA Authorization because the research protocol met the following criteria: (1) use or disclosure of the requested information involves no more than a minimal risk to the privacy of individuals; (2) the research could not practicably be conducted without the waiver; and (3) the research could not practicably be conducted without access to and use of the requested information.

## Conflict of Interest Statement

The authors have no conflicts of interest to declare.

## Funding Sources

This research was supported in part by the National Institutes of Health, including the National Cancer Institute (R01CA268030 [A.T.R., L.-S.C., M.P., and L.J.B.], UG3CA305777 [L.-S.C. and A.T.R.], and U19CA203654 [L.J.B., A.T.R., and L.-S.C.]), and the National Institute on Drug Abuse (K02DA065836 [A.T.R.], R01DA056050 [L.-S.C., A.T.R., and L.J.B.], and R44DA055161 [A.T.R.]), and the National Center for Advancing Translational Sciences (UL1TR002345 [A.T.R., L.-S.C., M.P., and L.J.B.]). This research was also supported by the Department of Defense (LC230570 [L.-S.C.]). This research was also supported in part by the Taylor Family Institute for Innovative Psychiatric Research (A.T.R.), the Washington University Department of Psychiatry Funding Program (A.T.R. and J.M.), and the Siteman Research Program Catalyst Award (A.T.R. and L.-S.C.). The funder had no role in the design, data collection, data analysis, and reporting of this study.

## Author Contributions

J.M.: conceptualized and designed the study, completed data analysis, drafted the manuscript. A.L., A.A., and T.R.: Facilitated study execution and data collection, reviewed and approved the final manuscript before submission. N.S.: Conceptualized and designed the study, facilitated study execution and data collection, reviewed and approved the final manuscript before submission. P.S.: conceptualized and designed the study, reviewed and approved the final manuscript before submission. M.C.P.: critically reviewed and provided revisions to the manuscript, reviewed and approved the final manuscript before submission. L.J.B.: conceptualized and designed the study, critically reviewed and provided revisions to the manuscript. L.-S.C.: conceptualized and designed the study, reviewed and approved the final manuscript before submission. A.T.R.: conceptualized and designed the study, provided guidance on data analysis, critically reviewed and provided revisions to the manuscript, and reviewed and approved the final manuscript before submission.

## Data Availability

The data that support the findings of this study are not publicly available due to privacy reasons but are available from the corresponding author upon reasonable request.
